# Comprehensive immunotherapy combined with intratumoral injection of zoledronate-pulsed dendritic cells, intravenous adoptive activated T lymphocyte and gemcitabine in unresectable locally advanced pancreatic carcinoma: a phase I/II trial

**DOI:** 10.18632/oncotarget.22974

**Published:** 2017-12-05

**Authors:** Yoshiki Hirooka, Hiroki Kawashima, Eizaburo Ohno, Takuya Ishikawa, Takashi Kamigaki, Shigenori Goto, Masashi Takahara, Hidemi Goto

**Affiliations:** ^1^ Department of Endoscopy, Nagoya University Hospital, Nagoya, Japan; ^2^ Department of Gastroenterology and Hepatology, Nagoya University Graduate School of Medicine, Nagoya, Japan; ^3^ Seta Clinic, Tokyo, Japan; ^4^ Department of Next Generation Cell and Immune Therapy, Juntendo University School of Medicine, Tokyo, Japan; ^5^ Medinet Medical Institute, MEDINET Co. Ltd., Yokohama, Japan

**Keywords:** immunotherapy, zoledronate-pulsed DCs, gemcitabine, advanced pancreatic carcinoma, EUS-guided FNA

## Abstract

Dendritic cell (DC)-based vaccines prepared using various antigen loading methods have been studied for cancer immunotherapy. The *in vivo* provocation of immunity by the direct injection of DCs without using tumor-specific antigens into tumors after apoptosis-inducing chemotherapy is more applicable. We previously reported that zoledronate-pulsed DCs (Zol-DCs) may induce tumor-antigen-specific CD8+ T cells by activating Vγ9γδT cells. In this report, we studied the feasibility, safety, and efficacy of a comprehensive immunotherapy involving the combined intratumoral injection of Zol-DC, gemcitabine (GEM) and αβT cells in locally advanced pancreatic carcinoma. Seven of 15 patients showed a stable disease (SD) and most of the patients showed long-term clinical responses. The FACT-BRM score was significantly higher in the patients with SD. Additionally the CD8+/Treg ratio significantly increased in SD patients after treatment. The median over-all survival and progression-free-survival of 15 patients were 12.0 months and 5.5 months, respectively. Patients with a pretreatment neutrophil/lymphocyte ratio (NLR) lower than 5.0 showed significantly longer survival. Even in an analysis limited to the patients with an NLR lower than 5.0, the patients whose CD8+/Treg ratio increased more than twofold tended to survive longer. In conclusion, the comprehensive immunotherapy using Zol-DCs, systemic αβT cells, and GEM may synergistically show a therapeutic effect on locally advanced pancreatic carcinoma. By using appropriate and precise biomarkers, such as NLR and CD8+/Treg ratio, the present comprehensive immunotherapy could be more beneficial for patients with pancreatic carcinoma.

## INTRODUCTION

Invasive pancreatic ductal carcinoma has a poor prognosis. Gemcitabine (GEM) improves the survival rate of advanced or recurrent pancreatic carcinoma but the 5-year survival rate is still 5% or lower [1, 2]. Locally advanced pancreatic carcinoma with celiac or suprameseteriac arterial invasion is considered unresectable and it is necessary to develop a new therapy. In clinical practice, various treatments are selected depending on the condition of the patients with locally advanced unresectable pancreatic carcinoma. Two randomized control studies in which the efficacy is compared between GEM alone and chemoradiotherapy have been carried out [3, 4]. In one study, the GEM-alone group showed a significantly prolonged survival than the Cisplatin + 5-FU combined with radiotherapy group [3]. On the other hand, better results were obtained in the GEM plus radiotherapy group than in the GEM-alone group in another study [4]. Thus, it is unclear which combination of various treatments provides better advantage for the patients with locally advanced unresectable pancreatic carcinomas.

Our previous study demonstrated that the combined therapy of intratumoral injection of OK-432-pulsed dendritic cells (DCs) and systemic injection of GEM and *ex vivo* expanded αβT cells might be synergistically effective and might have a therapeutic role in locally advanced pancreatic carcinoma by inducing tumor-antigen-specific cytotoxic T lymphocytes (CTLs) [5]. It is important that the cancer immunity cycle is completed, in order for the immunotherapy to exert its antitumor effect on malignancies. A neoantigen is a mutant antigen associated with genetic mutation accumulated in cancer cells and may elicit strong immune responses from T cells [6, 7]. In the first step, the neoantigen produced by tumorigenesis should be released and trapped by DCs for processing. A OK-432-pulsed DCs vaccine combined with GEM for patients with pancreatic carcinoma has been shown to induce tumor-antigen-specific CTLs in one of five patients in a previous study [5]. Thus, the combination of injection of DCs into the tumor and the cytotoxic effect of GEM may promote antigen uptake into DCs in the first step of the cancer immunity cycle. Our previous data also suggest that immunotherapy with tumor-antigen-pulsed immature DCs with zoledronate (Zol-DCs) results in the activation of Vγ9γδ T cells and the induction of CD40L on Vγ9γδT cells [8]. Activated Vγ9γδ T cells secrete Th1-cytokines such as interferon (IFN) - γ, which promotes the expansion of tumor-antigen-specific CD8 + T cells by immature DCs pulsed with tumor antigens. Osada et al. reported the functional analysis of DCs pulsed with zoledronate or OK-432. In immature DCs, cell surface marker analysis suggests that Zol-DCs essentially have equivalent function to OK-432-DCs [9]. These findings suggest that Zol-DCs potentially elicit antigen-specific immunity similarly to OK-432-pulsed DCs.

In this study, we examined the safety, feasibility, and therapeutic effect of a comprehensive cancer immunotherapy by the intratumoral administration of Zol-DCs and the systemic injections of αβ T cells and GEM in patients with locally advanced unresectable pancreatic carcinoma.

## MATERIALS AND METHODS

### Study design

This study was a single-arm, open-label phase I-II study of patients with locally advanced unresectable pancreatic carcinoma. The aim of this study was to evaluate the efficacy and safety of the combination of GEM and immunotherapy, including both Zol-DCs and αβT cells, as the first-line therapy for these patients. The primary efficacy endpoint was over-all survival (OS), and the secondary endpoints included one-year survival rate, progression free survival (PFS), cytoreductive effect, levels of tumor markers, and quality of life (QOL). We also evaluated immunological responses as well as safety.

The study protocol was approved by the Research Ethics Committee of Nagoya University Graduate School of Medicine and written informed consent was obtained from all the participants. This clinical study was registered in the University Hospital Medical Information Network Clinical Trials Registry (UMIN-CTR) with the trial number UMIN000000769.

### Patients

This study was conducted between June 2007 and March 2015 using 15 patients with histopathologically confirmed measurable locally advanced unresectable pancreatic carcinoma that was not previously treated with chemotherapy or radiotherapy. All patients were confirmed having as pancreatic ductal adenocarcinoma by endoscopic ultrasound-guided fine needle aspiration (EUS-FNA). Other key inclusion criteria were as follows: 20 to 75 years of age, a performance status (PS) of 0 to 2 on the Eastern Cooperative Oncology Group (ECOG) scale, and adequate bone marrow, liver and renal functions. The key exclusion criteria were as follows: major medical history of interstitial pneumonia, cardiovascular disease, or cerebral vascular disease, active autoimmune disease, pregnancy or breast-feeding, and positivity for an antibody to HIV or HTLV-1 within the past 6 months.

### Preparation of Zol-DCs and αβT cells

Peripheral blood (PB) was isolated using leukapheresis. Peripheral blood mononuclear cells (PBMCs) were isolated by density gradient centrifugation using lymphoprep (Nycomed, Oslo, Norway). These cells were allowed to adhere to tissue culture flasks with AIM-V medium (Gibco, Gaithersburg, MD) for 1 h at 37°C. Non-adherent cells were used for preparing αβT cells by culturing with an immobilized monoclonal anti-CD3 antibody (Jansen Pharmaceutical K.K, Tokyo) in the presence of recombinant human IL-2 and autologous plasma for 14 days [10]. Adherent cells were cultured in a serum-free AIM-V medium containing recombinant human IL-4 (Osteogenetics GmbH., Wuerzburg, Germany) and recombinant human granulocyte/macrophage colony-stimulating factor (GM-CSF) (Immunex, Richmond, CA) for 7 days to generate immature DCs, which were further cultured with zoledronate (Chugai Pharmaceutical Company, Tokyo, Japan) for 24 h to obtain Zol-DCs [8]. The Zol-DCs were cryopreserved until the day of administration. Surface molecules expressed by the Zol-DCs were determined by flow cytometry. The cells defined as Zol-DCs in this study were CD14-, HLA-DR+, HLA-ABC+, CD80+, CD86+, CD40+, CD54+, and CD83 (weak). Fresh αβT cells were prepared every injection as described above; it was composed of more than 90% CD3+T- cells.

### Treatment

GEM, at a dose of 1000 mg/m^2^ of body-surface area, was administered by 30-minute intravenous infusion on Day 1, followed by EUS-FNI of Zol-DCs into pancreatic tumors and 30-minute intravenous infusion of αβT cells on Day 4 of each 14-day cycle, which was planned to be conducted in 12 cycles for each patient. During the study period, some drugs and treatments were prohibited: anticancer drugs other than GEM, biological response modifiers including cytokines, systemic steroids, unapproved medications, and radiotherapy.

### Clinical monitoring

At the start of each cycle, we assessed the patient's general status, PS and QOL using the functional assessment of cancer therapy-biologic response modifier (FACT-BRM) [11], and safety using the Common Terminology Criteria for Adverse Events (CTCAE) version 3.0. We also carried out complete blood count and chemical tests every 2 weeks, evaluated the expression levels of serum tumor markers including the carcinoembryonic antigen (CEA), carbohydrate antigen 19–9 (CA19-9), and pancreatic cancer-associated antigen (DUPAN2), and performed diagnostic imaging every 4–6 weeks. Tumor responses were determined using the Response Evaluation of Criteria in Solid Tumors (RECIST). In cytoreductive effect analysis, a summary statistic of tumor control probability was estimated on the basis of percentages of complete response (CR), partial response (PR), long stable disease (SD), sustained SD (SD longer than six months), and progressive disease (PD) which were evaluated using RECIST. Moreover, immunological tests were evaluated at Cycles 1, 6, and 12, and immunohistochemical analysis was carried out examined when tumor samples were obtained by biopsy during endoscopy. Slides of tumor samples were preparedin the Department of Medical Technique, Clinical Laboratory, Nagoya University Hospital, and the intensity and localization of staining were evaluated. The levels and distribution of the expression were qualitatively estimated and scored as − (0), + − (1–2), + (< 50), and ++ (≥ 50) according to the number of positive cells in a field of a 40 times objective.

### Statistical analyses

OS, one-year survival rate, and PFS were estimated using the Kaplan-Meier method. QOL was analyzed using the linear mixed effects model. All statistical analyses were performed using JMP, version 11.2.0 for Microsoft Windows 7 (SAS, Cary, NC, USA).

## RESULTS

### Patient characteristics

Between June 2007 and March 2015, written consent was obtained from 16 patients, among whom 15 patients were enrolled in this study, and one patient who met one of the exclusion criteria was excluded. The clinical characteristics of the patients are shown in Table 1. The median age of the 15 patients (10 males and 5 females) was 66 years (range: 49–75 years). Eleven patients had tumor in the pancreatic body, three in the pancreatic head, and one in the pancreatic tail, and all of them showed no metastasis.

**Table 1:** Patient characteristics and data on immunotherapy performed

Patient Characteristics			Immunotherapy Performed			Crinical Response		
Patient	Age/Sex	Location	No. Courses	No. DCs Administered (×10^6^ cells) (Mean)	No. abT cells Administered (×10^6^ cells) (Mean)	Progression Free survival (months)	Overall Survival (months)	Clinical Response (After 12th Infusion)
1	64/F	body	11	3.1	7.0	4.5	11.5	PD
2	69/M	head	8	7.7	5.7	3.6	17.7	PD
3	49/M	body	12	11.0	6.2	6.3	10.0	SD^*^
4	69/F	body	12	19.3	5.7	58.5	73.4	SD^*^
5	67/M	body	1	18.4	6.8	5.7	7.6	PD
6	62/M	body	5	14.4	6.7	2.5	6.4	PD
7	67/M	body	12	16.2	6.2	17.7	37.7	SD^*^
8	75/M	body	12	17.7	6.2	11.5	12.4	SD^*^
9	63/M	body	12	25.0	4.2	12.9	38.6	SD^*^
10	66/F	tail	10	39.0	5.3	3.6	5.0	PD
11	66/M	head	5	34.0	5.4	1.7	15.0	PD
12	55/F	head	12	20.3	7.2	21.3	26.8	SD^*^
13	58/F	body	9	17.9	4.8	2.7	7.5	PD
14	54/M	body	12	18.2	6.0	5.5	8.6	PD
15	71/M	body	5	23.0	5.0	2.1	2.1	SD

^*^ longer than 6 months SD

### Safety and toxicity

A total of 33 adverse events were recorded during the study period (Table 2). Grade 3 adverse events occurred in four patients, in which those in two patients were likely related to the GEM administrated. No dose limiting toxicity occurred in this study. Patient 15 (classified as SD) was withdrawn from the study, because of attempted suicide. None of the remaining patients withdrew from the study because of adverse events. Irrespective of the number of DCs administrated, Patient 8 experienced grade 1 fever twice and grade 3 fever once 24 to 48 hours after DC injection, which were considered to be probably related to the immunotherapy, and were responsive to common antipyretics.

**Table 2:** Adverse events

	Grade				Total
	1	2	3	5	
Anorexia	4	8			12
Leukocytopenia		1	1		2
Weight loss		2			2
Nausea	2				2
Decreases in Hb level			1		1
Anemia	2				2
Urticaria (hives, welts, wheals)		1			1
Liver dysfunction/failure (clinical)	2				2
Allergic reactions/hypersensitivity	1				1
Diarrhea	1				1
Pancreatitis^*^		1			1
Fever^*^	2		1		3
Rash^*^	1				1
Infection-Others^**^ (Intraabdominal abcess)			1		1
Suicide attempt^***^				1	1

^*^ immunotherapy and/or EUS-FNI-related adverse events

^**^ EUS-FNI-related adverse event

^***^ Non-immunotherapy and EUS-FNI-related adverse event

### Clinical outcomes

Tumor responses were evaluated using RECIST. Seven of the 15 patients demonstrated some good responses in this clinical study: sustained SD (longer than 6 months) in six patients and SD in one patient. PFS was defined as the time from registration to the first observation of PD or death due to any cause. In the 15 patients, the PFS was in the range from 1.7 to 58.5 months and the OS was between 2.1 months and 73.4 months, as shown in Table 1. The median survival time (MST) was 11.5 months. In more detail, three (Patients 4, 7, and 9) of seven SD patients survived longer than three years. One (Patient 8) of the seven SD patients underwent surgery removal of cancer after the 12th treatment cycle.

### QOL assessment by FACT-BRM

**Figure 1:**
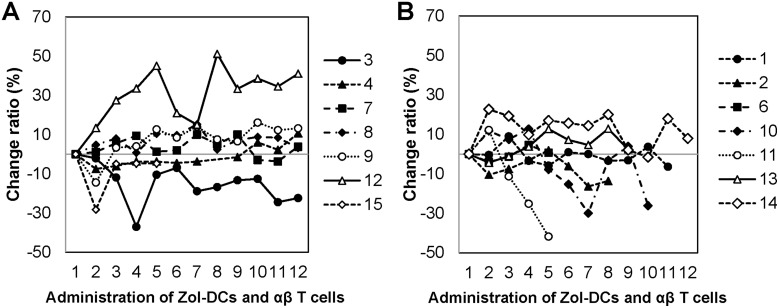
Change ratio in FACT-BRM score during administration (**A**) Change ratio in FACT-BRM score after injection in SD group. (**B**) Change ratio in FACT-BRM score after injection in PD group.

FACT-BRM instrument was used to evaluate the results of this clinical study (Figure 1). FACT-BRM score changes after injection in different groups were assessed using the linear mixed effects model. The FACT-BRM scores of both groups tended to increase with the course of treatment. The rate of change in FACT-BRM score after injection was significantly higher in the SD group than in the PD group (*p* = 0.038).

### Immunological responses

**Figure 2:**
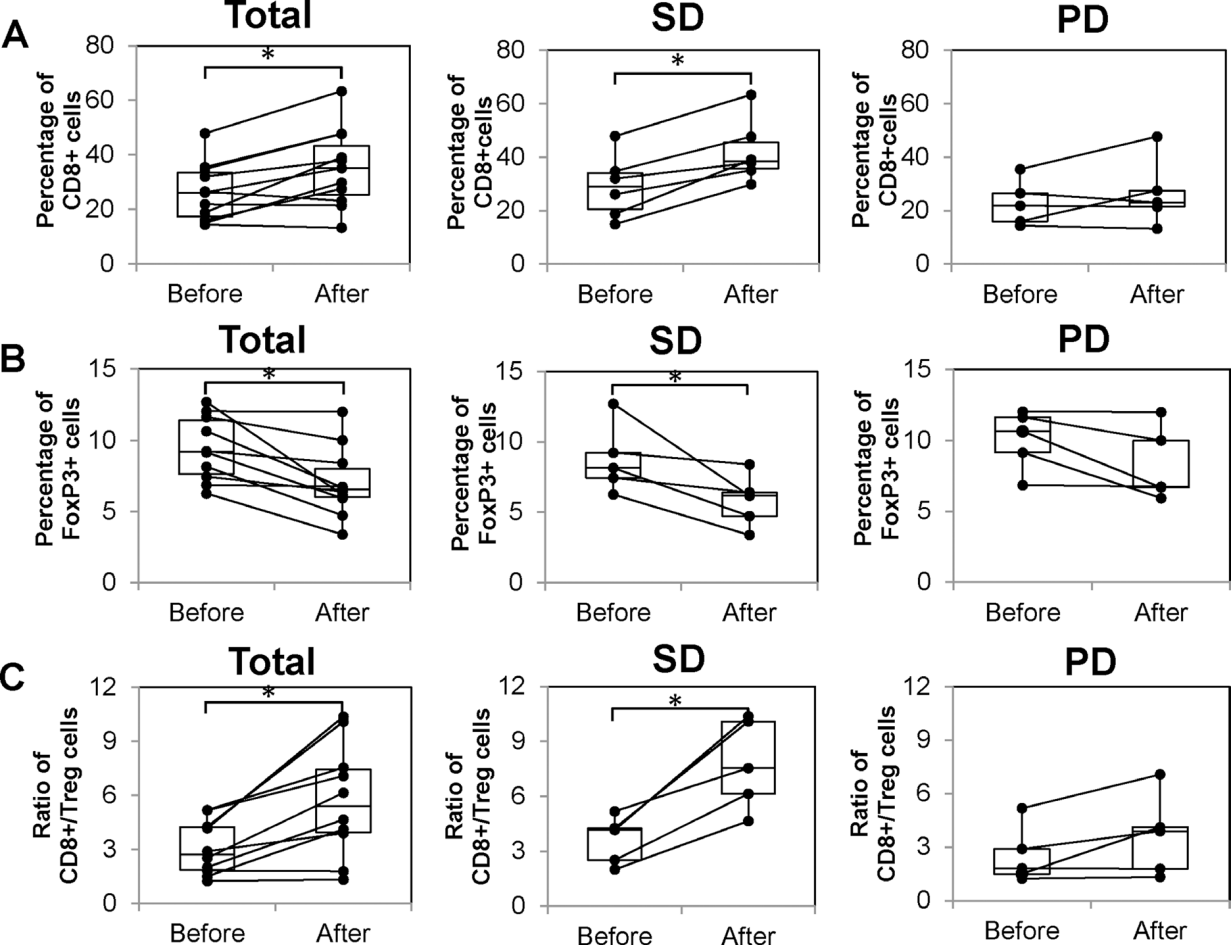
Immunological responses before and after administration The left graphs show results of all patients that were measurable before and after administration. The middle graphs show the results of only SD patients and the right graphs show those of PD patients only. (**A**) Number of CD8+ T cells before and after 6th administration. (**B**) Number of FoxP3+ T cells before and after 6th administration. (**C**) Ratio of CD8+/Treg cells before and after 6th administration.

As shown in Figure 2, T cell subsets contained in peripheral blood before and after treatment (just before the 6th administration) were evaluated in 11 patients who were able to receive six cycles of treatment. As a result, the number of CD8+ T cells significantly increased after treatment (26.2 ± 3.2 vs 35.0 ± 4.3; *p* = 0.003). The number of T cells was highest in the SD group than in the PD group, although only the SD group showed a significant increase (29.1 ± 4.9 vs 42.1 ± 4.9; *p* = 0.002; Figure 2A). On the other hand, the number of regulatory T cells (Treg; CD4+, FoxP3+) significantly decreased after treatment (9.4 ± 0.7 vs 7.0 ± 0.8; *p* = 0.005). This decrease was significant in the SD group (8.8 ± 1.1 vs 5.8 ± 0.8; *p* = 0.046), but not in the PD group (*p* = 0.087; Figure 2B). The ratio of the number of CD8 + T cells to that of Treg cells (hereafter CD8+/Treg ratio) was significantly increased after the treatment (3.1 ± 0.5 vs 5.7 ± 1.0; *p* = 0.004). This increase was significant in the SD group (3.6 ± 0.6 vs 7.8 ± 1.1; *p* = 0.007), but not in the PD group (*p* = 0.096) (Figure 2C).

### Immunohistological staining

**Figure 3:**
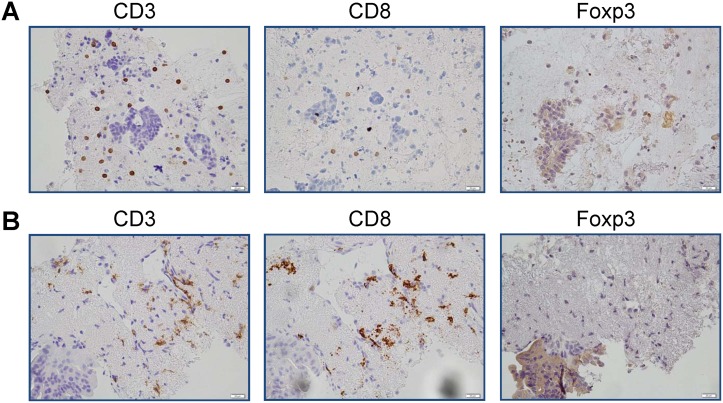
Results of immunohistological staining of samples from SD patient Results of immunohistological staining for (**A**) Patient 7 before administration and (**B**) Patient 7 after administration. Many CD8+ T cells infiltrated into the tumor tissue after treatment.

Immunohistological staining was carried out for all biopsy tissue samples obtained by endoscopy. Figure 3 shows results of the immunohistological staining of a sample from Patient 7 in the SD group. Many CD8 + T cells infiltrated into the tumor tissue after treatment, whereas no infiltration of FoxP3+ cells was observed. However, there was no correlation between the infiltration of CD8+ T cells into the tumor tissue and the clinical outcome. Furthermore, there was no clear correlation between the increase and decrease in the number of lymphocytes in the peripheral blood.

### Assessment of survival

**Figure 4:**
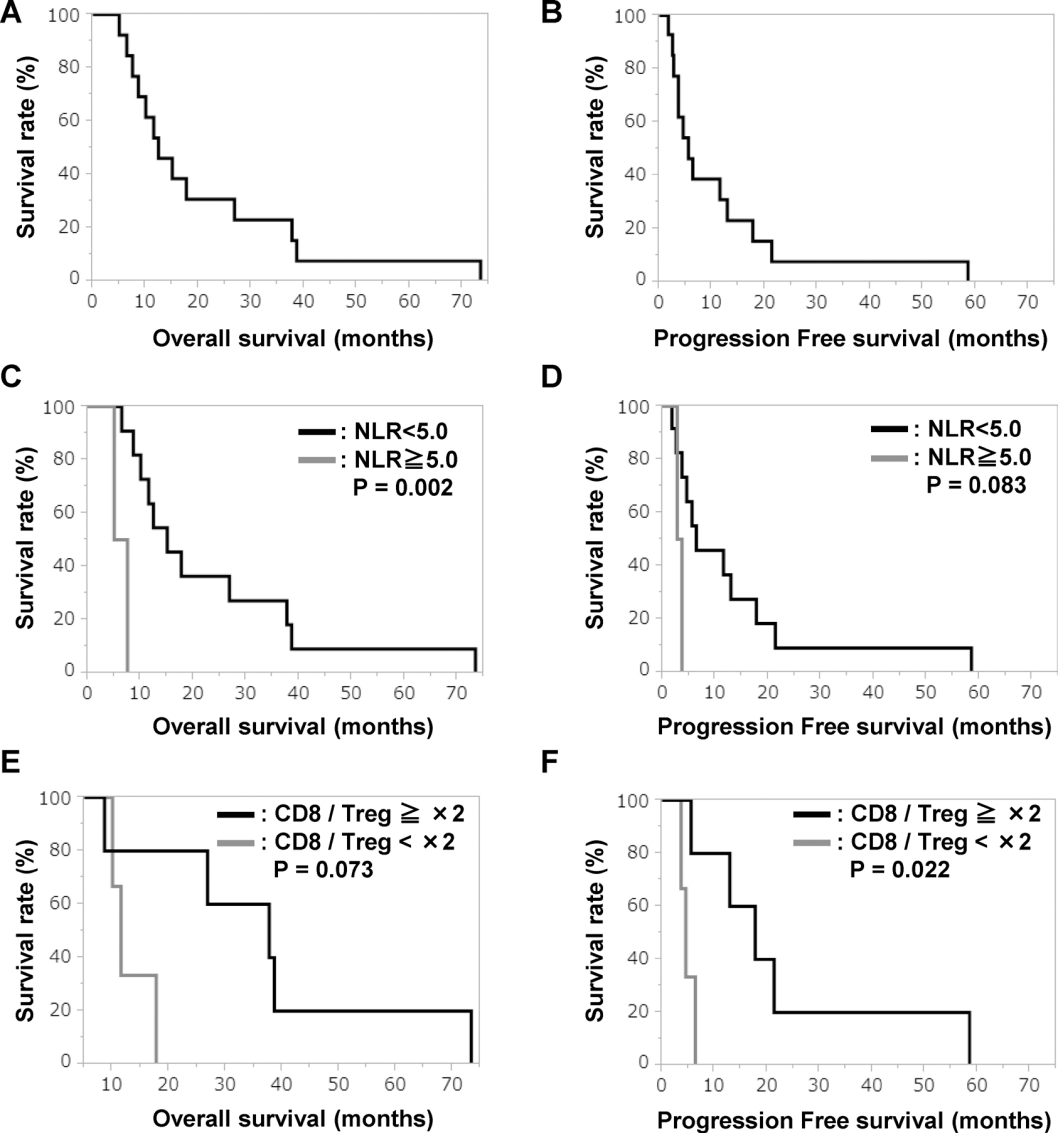
OS and PFS Kaplan-Meier curves of (**A**) OS for all patients, (**B**) PFS for all patients, (**C**) OS for patients with NLRs lower than 5.0 and higher than 5.0, (**D**) PFS for patients with NLRs lower than 5.0 and higher than 5.0, (**E**) OS with respect to rate of change in CD8+/Treg ratio, and (**F**) PFS with respect to rate of change in CD8+/Treg ratio.

As shown in Figure 4A, the median follow-up period for survivors was 12.0 months (range, 2.1–73.4 months). The PFS was 5.5 months (range, 1.7–58.5 months) (Figure 4B). We analyzed whether age, sex, tumor marker, white blood cell counts and the ratio of the number of neutrophils to that of lymphocytes [hereafter neutrophil lymphocyte ratio (NLR)] affect OS and PFS. As a result, the OS of patients with NLRs lower than 5.0 extended significantly compared with that of patients with an NLR of 5.0 or higher, and the PFS of patients with NLRs lower than 5.0 tended to extend (Figure 4C and 4D) (23.5 months vs 6.3 months, 13.4 months vs 3.2 months) (*p* = 0.002, *p* = 0.083). In the analysis of the patients with NLRs lower than 5.0 before treatments, those who showed a more than two fold increase compared with patients who showed no change in the CD8+ / Treg ratio, the OS tended to extend and PFS significantly extended (Figure 4E and 4F) (37.0 months vs 13.1 months, 23.2 months vs 4.8 months) (*p* = 0.073, *p* = 0.022).

## DISCUSSION

In the case of DC-based cancer vaccines pulsed with various tumor-associated antigens, insufficient clinical responses have been reported, despite the superior immune responses in terms of delayed-type hypersensitivity (DTH) [12]. Previously, we studied a novel immunotherapy in which intratumor injection of OK-432-pulsed DCs is combined with the systemic intravenous administration of αβT cells and GEM for locally advanced unresectable pancreatic carcinoma [5]. In the phase I study, it was found that an immune response to the protein antigens extracted from the tumor was elicited after the treatment. We also employed Zol-DCs to develop vaccines that target various tumor-associated antigens (TAAs) and characterized them in various clinical settings, including intratumoral injection of DCs [13]. In this study, we investigated the safety and antitumor effect of a comprehensive cancer immunotherapy by the intratumoral injection of Zol-DCs into locally advanced unresectable pancreatic carcinoma, combined with systemic αβT cell therapy and GEM chemotherapy.

Fifteen patients with unresectable locally advanced pancreatic carcinomas were enrolled in this study. Seven of them showed SD and most of these seven patients showed favorable long-term clinical responses. Regarding adverse events, most of them were of Grade 1 or 2; thus, it was considered that the present comprehensive cancer immunotherapy was well tolerated. We have reported that αβ T cell therapy and DC-based vaccines can be applied safely for the immunotherapy of various solid tumors [14]. For the adverse events of EUS-FNA, the percentages of perforation, bleeding, and infection were all 1.3%, and the risk of pancreatitis was reported to be about 1 to 2% [15]. In this study, grade 1 pancreatitis occurred in one patient which was treated conservatively. Among 75 EUS-FNA biopsies previously reported, one resulted in abdominal abscess as a serious adverse event (1.3%), which required surgical drainage [16]. In patient 5, intraperitoneal abscess formed which was considered to be related to the first intratumoral injection of DCs. Drainage was necessary for this patient and this research was terminated. Fortunately, the patient recovered after drainage and antibiotic therapy. In this combination therapy, utmost care should be taken for adverse events of Zol-DCs endoscopic intratumoral injection. It may be difficult to predict the occurrence of an intraperitoneal abscess because the target tumor tends to be fragile during the course of treatment. Careful targeting of the tumor and injection of Zol-DCs with real-time monitoring the leak as a hyperechoic ultrasound image may prevent or minimize the occurrence of this event.

In GEM-based chemotherapy, QOL is often maintained in the treatment of pancreatic carcinoma. In gemcitabine plus irinotecan treatment of inoperable pancreatic carcinoma, 13 of 15 patients showed QOL improvement and the remaining two patients showed no improvement [17]. For SD patients, QOL improvement was observed in 4 out of 7 patients. Moreover, the FACT-BRM score of the SD patients was significantly higher than that of the PD patients. The present data suggest that our comprehensive cancer immunotherapy could contribute to the improvement of the QOL of the patients with locally advanced pancreatic carcinoma.

The results of immunomonitoring of peripheral blood revealed that the present comprehensive cancer immunotherapy showed a significant increase in CD8+ T cell count and a significant decrease in Treg ratio, particularly in SD patients with locally advanced pancreatic carcinoma. Additionally, a significant increase in CD8+/Treg ratio was observed in SD patients. In our previous study, the concurrent αβ T cell therapy and DC-based vaccine therapy successfully increased the number of CD8+ T cells in the peripheral blood of patients with various types of cancer [18]. In some patients, the analysis of immunohistochemical staining showed both the increase in the number of CD8+ T cells and the decrease in that of Treg cells in the tumor sites as well as in the peripheral blood. In the present comprehensive cancer immunotherapy, immunological monitoring of peripheral blood may indicate immune fluctuation in the local tumor site.

The OS of the patients with locally advanced pancreatic carcinoma who received the combined cancer immune cell therapy was 12.3 months, and it was almost equivalent to the OS of the patients who received GEM-based chemoradiotherapy. The present comprehensive immunotherapy could potentially be one of promising treatment against locally advanced pancreatic cancer, because the 2-year survival rate was 30.8%, although the 2-year survival rates were reported to be 5–12% in the locally advanced pancreatic cancer patients received with GEM only or chemoradiotherapy [3, 4]. It was reported that NLR may be a predictor of the efficacy of various cancer immunotherapies, such as those using immune checkpoint inhibitors [19, 20]. NLR has been reported to be a marker of systemic inflammatory response and cancer-related inflammation has also been reported to be associated with progression and survival in various solid tumors [19, 20].

The present study demonstrated that the prognosis of patients with an NLR of 5 or higher at the start of treatment is significantly worse than that of patients with an NLR lower than 5. Therefore, in future clinical trials, NLR ≥ 5 should be an exclusion criterion for patient registration. Even for the patients with pretreatment NLR > 5 in locally advanced pancreatic cancer, when NLR decreases to less than 5 after other chemotherapy or radiotherapy, it is also necessary to further consider whether the present comprehensive immunotherapy will be indicated or not. In the analysis of CD8+/Treg ratio after the 6th injection of αβ T cells and immature DCs, the OS of the patients whose CD8+/Treg ratio increased more than twofold that before treatment was significantly better than the OS of patients with less than twofold increase. The CD8+/Treg ratio has been reported to be a useful candidate marker for predicting prognosis associated with cancer immunity [21, 22]. The present results indicate that the CD8+/Treg ratio could be a useful biomarker for determining whether to continue treatment or not. It could also be essential for long-term survival that the CD8+/Treg ratio increased more than twofold during treatment. In order to increase the CD8+/Treg ratio, it should be necessary to transfer more CD8+ T cells adaptively or to combine other treatments to reduce Tregs.

In conclusion, the comprehensive cancer immunotherapy using Zol-DCs may be beneficial for patients with locally advanced pancreatic carcinoma when used on the basis of appropriate biomarkers, such as NLR and CD8+/Treg ratio.

In future clinical trials, we have to solve two problems. One is to select the optimal anticancer agent to be used in combination, because in advanced or recurrent pancreatic carcinoma, combination chemotherapy superior to GEM alone, such as GEM / nab-PTX and FOLFIRINOX, has also been developed. Secondly, it is necessary to examine whether the antitumor effect is enhanced in combination with immune checkpoint inhibitors.
